# The contribution of forward masking to saccadic inhibition of return

**DOI:** 10.3758/s13414-018-1490-2

**Published:** 2018-03-08

**Authors:** David Souto, Sabine Born, Dirk Kerzel

**Affiliations:** 1 Department of Neuroscience, Psychology and Behaviour, University of Leicester, Lancaster Road, Leicester, LE1 9HN UK; 2 Faculté de Psychologie et des Sciences de l’Éducation, Université de Genève, Geneva, Switzerland

**Keywords:** Inhibition of return, Forward masking, Saccadic eye movements, Reaction times, Orientation-tuning, Contrast gain control, Contrast adaptation

## Abstract

**Abstract:**

*Inhibition of return* is the name typically given to the prolonged latency of motor responses directed to a previously cued target location. There is intense debate about the origins of this effect and its function, but most take for granted (despite lack of evidence) that it depends little on forward masking. Therefore, we re-examined the role of forward masking in inhibition of return. Forward masking was indexed by slower saccadic reaction times (SRTs) when the target orientation repeated the cue orientation at the same location. We confirmed effects of orientation repetition in the absence of an attentional bias when cues were presented on both sides of fixation (bilateral presentation). The effect of orientation repetition was reduced with high target contrast, consistent with a low-level origin such as contrast gain control in early visual areas. When presenting cues on only one side of fixation (unilateral presentation), we obtained inhibition of return with longer cue-target intervals and facilitation with targets presented shortly after the cue. The effect of orientation repetition was reduced when facilitation was observed, but was as strong as with bilateral cues when inhibition of return was observed. Therefore, forward masking may contribute to the inhibition of return effect by delaying reaction times to repeated features at the same location, but is not a principal cause of inhibition of return; in agreement with previous views.

**Significance statement:**

The saccadic inhibition of return effect is a reaction-time cost when responding to a pre-cued location. Additional object updating costs are typically invoked to explain reaction-time costs observed when cue and target have the same shape. Yet, lower-level, forward masking of the target by the cue can not be ruled out. Importantly, we show an effect of orientation repetition that is consistent with low-level forward masking rather than object updating costs and that does not interact with inhibition of return.

## Introduction

Our ability to react to a stimulus, such as a red light at a pedestrian crossing, depends on previous stimulation. Simple cueing paradigms have been extensively used to investigate the effects of preceding stimuli on subsequent action and perception (Fecteau & Munoz, 2007). Here we focus on the effect of repeating a stimulus feature on saccadic reaction times. Since we move our eyes around continuously to explore the visual world for fixation periods of around 250 ms, the effect of briefly presented visual patterns on subsequent reactions to same or different objects is of special relevance to understanding eye movement decisions.

Among various cueing effects, inhibition of return (IOR) has a prominent place. In a typical involuntary cueing paradigm, a cue precedes the target unpredictably at the same or at a different location, with different stimulus onset asynchronies (SOAs) separating the two events. Under some conditions responses show a biphasic profile: cues presented shortly before the target (50–250 ms) facilitate reaction times to targets at the cued location, whereas cues presented long before the target (often >300 ms) delay reaction times (for reviews see Chica, Martín-Arévalo, Botta, & Lupiáñez, 2014; Klein, 2000). The latter delay has been called inhibition of return, alluding to the idea that reorienting to the cued peripheral location is inhibited in favor of uncued locations (Posner & Cohen, 1984). However, the IOR effect is likely to have several origins, with sensory, motor, and attentional processes contributing (Lupiáñez, 2010). Here, we examine the contribution of forward pattern masking to the IOR effect observed in a peripheral cueing paradigm.

Forward pattern masking (henceforth forward masking) is a reduction in a pattern’s visibility when it is preceded by a similar pattern (Öğmen & Breitmeyer, 2006). A contribution of forward masking to the IOR effect was dismissed as early as Posner and Cohen’s (1984) seminal paper, as it was thought to be too short lived. More recently, interest in sensory components of IOR has known a resurgence in light of neurophysiological findings. Visual-motor neurons of the superior colliculus (a mid-brain structure linked to IOR) are suppressed after repeated stimulation, likely because they receive an attenuated feedback from cortical visual areas (Dorris, Klein, Everling, & Munoz, 2002). Moreover, the behavioral observation that the repetition of the same shape in cue and target can delay reaction times also suggests a contribution of sensory factors to IOR (Patel, Peng, & Sereno, 2010; Red, Patel, & Sereno, 2012).

The effects of feature and shape repetition are central to understanding the possible contribution of forward masking. Earlier findings showed that inhibition of responses by the repetition of the same color occurs for central but not for peripheral cues (Fox & de Fockert, 2001; Kwak & Egeth, 1992; Law, Pratt, & Abrams, 1995). Shape repetition, however, delays reaction times when peripheral cues are used (Riggio, Patteri, & Umilta, 2004). The shape-similarity effect is maximal with a 200 ms-SOA, and disappears within 500 ms. There is a possibility that shape-based effects may be mediated by feature-level effects (e.g., orientation similarity), which would indicate a lower-level sensory origin (Patel et al., 2010; Riggio et al., 2004). Although some authors attribute the costs of repeating the same stimulus at the same location to sensory adaptation (Hilchey, Klein, & Satel, 2014), other appeal to higher-level constructs, such as the updating of object-files (Lupiáñez, 2010). Put simply, object-updating accounts claim that it takes more time to decide whether cue or target is presented when the two are very similar and presented in close succession, which delays responses to the target (e.g., Schönhammer & Kerzel, 2017). In contrast, a sensory adaptation account attributes delayed reaction time to the attenuated response of feature detectors after prolonged or repetitive stimulation. For instance, cells in the macaque cortex are significantly less sensitive to a pattern when it is repeated with the same orientation compared to a cross-oriented one, even though the first presentation may have only been brief (Muller, Metha, Krauskopf, & Lennie, 1999). Sensory adaptation may in turn lead to forward masking effects and delayed saccadic responses by reducing the amount of sensory evidence available to reach a perceptual or motor decision. An important difference between accounts is that the sensory adaptation account would depend critically on the target signal strength and operates on individual features (e.g., orientation difference), whereas in the object-updating account, object-similarity is the main determinant, irrespective of signal strength.

For the sake of parsimony, before appealing to higher-level constructs (e.g., object updating), one should exclude lower-level effects. To this purpose, we aimed at delineating the contribution of lower-level forward masking to the saccadic IOR effect. We started by looking at responses to target gratings (low contrast targets in Experiment 1; a range of target contrasts in Experiment 2) that were preceded by bilateral high-contrast cue gratings with varying orientation relative to the target. Many physiological and psychophysical studies have used pattern orientation to investigate forward masking and sensory adaptation (e.g., Foley & Boynton, 1993; Muller et al., 1999), facilitating the interpretation of the findings. By first testing feature-repetition effects with bilateral cues we are able to isolate effects of the cue gratings on sensory processing of the target, because the bilateral presentation does not bias decisions to one side or the other. Any forward masking effect is therefore uncontaminated by attentional or response bias, and represents a baseline against which to compare the IOR effect (or facilitation; Experiment 3) induced by presenting the cue unilaterally. Thus, we will describe to which extent low-level forward masking contributes to IOR.

## Methods

Twenty-eight students (18–33 years old) from the University of Geneva participated in Experiment 1 for course credit; 14 (18–25 years old) took part in Experiment 2; 25 (18–29 years old) took part in Experiment 3a; 24 (18–31 years old) took part in Experiment 3b. All participants had normal or corrected-to-normal vision. The experimental protocol was approved by the ethics committee of the *Faculté de Psychologie et des Sciences de l’Education* and followed the guidelines of the Declaration of Helsinki.

### Materials

For all experiments except Experiment 3b, stimuli were generated by a ViSaGe graphics card (Cambridge Research Systems Ltd., Rochester, UK) and displayed on a 100-Hz CRT screen (Mitsubishi Diamond Pro 2070SB), with a resolution of 1,024 x 768 pixels. Experiments took place in a dimly lit room. Observer’s head was maintained fixed by a chin-forehead rest 67 cm away from the screen. Eye position was recorded by a video-based eye tracker with a sampling frequency of 250 Hz (CRS High Speed Video Eyetracker, Cambridge Research Systems Ltd., Rochester, UK). The eye tracker was calibrated at the beginning of each session by presenting nine points in a pseudo-random order covering the display area.

In Experiment 3b, the PsychToolbox for MATLAB (Brainard, 1997) was used to display the stimuli and the Eyelink 1000 (SR-Research, Osgoode, Ontario, Canada) was used to track eye movements. The refresh rate of the monitor was 85 Hz instead of 100 Hz.

### Stimuli and procedure

#### Experiment 1: Bilateral cue presentation

**Fig. 1:**
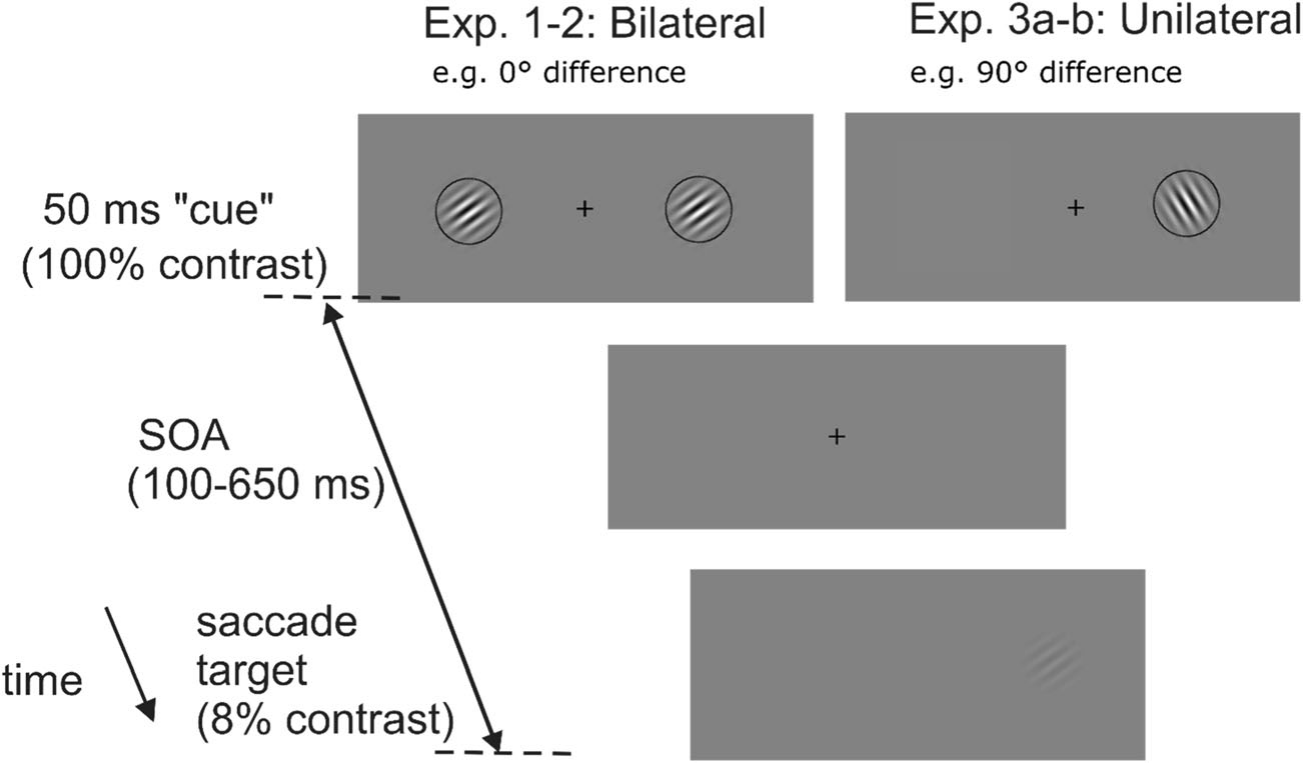
Stimuli and trial sequence for all experiments. In Experiments 1 and 2 (bilateral presentation), a 100% contrast Gabor was presented on both sides of the fixation cross for 50 ms. In Experiments 3a and 3b (unilateral presentation), the cue appeared randomly left or right of fixation. After an SOA of 100–650 ms (SOA range varying across experiments), the saccade target, a Gabor at 8% contrast (varying contrast in Experiment 2), was shown on the left or right side. Our primary interest was the effect of orientation difference between cue and target gratings on saccadic reaction times (SRTs). The difference is 0° (i.e., orientation repetition) with the cue shown in the left screen and 90° with the cue shown in the right screen

Stimuli and procedure in Experiment 1 are illustrated in Fig. 1. The background was light gray, with a luminance of 66 cd/m^2^. At the start of a trial, a foreperiod of 800–1,200 ms preceded the presentation of the cue. Cues were composed of high contrast (100% Michelson contrast) Gabor patches surrounded by a black circle. The black circle had a line width of 0.2° of visual angle (dva) and a diameter of 3 dva. The Gabor patches were composed of a sine-wave grating with a spatial frequency of 2 cycles/dva multiplied by a Gaussian with a standard deviation of 0.6 dva. Cues were presented at 5 dva to the left and right of a fixation cross for 50 ms. Circles were presented without Gabors in one-fourth of the trials, allowing for comparison with more classic cue types. The cue was followed, after a variable stimulus onset asynchrony (SOA: 100, 250, 450, or 650 ms), by a low contrast Gabor (8% Michelson contrast, same spatial frequency and SD as the cue Gabors), referred to as target, that was presented until a saccadic response was recorded. The phase of the sine-wave was the same in cue and target Gabors, but randomized from trial to trial. The orientation of the sine-wave in the cue Gabor patch was random (from 0° to 180°), as was the location of the target (left, right). The main stimulus variable of interest was the difference in orientation between cue and target Gabor patches. The target could be rotated relative to the cue by 0°, ±45°, or 90°.

We used a within-subjects design, with SOA as a blocked variable, while cue type (three cue-target orientation differences, plus the circle-only cue) and target location were randomly interleaved within a block of trials. There were 80 trials per condition (with -45° and +45° orientations pooled together). We balanced block order (SOA) according to a 2 x 2 Latin square design. The experiment was run in two sessions and each trial was started by observers pressing a designated button on a response box.

Observers were instructed to make a saccade to the second grating as soon as it appeared and to ignore the first event. They received error feedback whenever a blink occurred during the trial, the saccade was triggered too soon (<100 ms), too late (>700 ms), or landed further than 2 dva from the target location. Otherwise, fixation had to be maintained within 2 dva of the fixation dot.

#### Experiment 2: Target contrast manipulation

As in Experiment 1 we used a bilateral cue presentation, but only the three shortest SOAs were used: 100, 250, and 450 ms. The contrast of the target was varied in different blocks (8%, 20%, or 60% Michelson contrast), and we randomized block order by using a Latin square, whereby the number of different transitions between conditions was balanced. Only the 0° and 90° orientation difference conditions were run.

#### Experiment 3a: Interaction with inhibition of return

The design and procedure were as in Experiment 1 with the following exceptions: only one cue Gabor was presented on one side. Also, only the 0° and 90° orientation difference conditions were run. Cue types (two cue-target orientation differences), cue location, and target location were randomly interleaved within a block of trials.

#### Experiment 3b: Interaction with facilitation

This experiment complemented Experiment 3a by testing for a facilitation effect at earlier SOAs and by testing the effect of a circle-cue in a separate block of trials. The three SOAs were 50, 100, and 250 ms. Target gratings had 0° or 90° orientation relative to the randomly oriented cue. In the baseline condition, a circle cue was presented alone, the target being a randomly oriented grating. SOA and cue type (circle+Gabor vs. circle-only cue) were therefore blocked variables. Block order was balanced across participants by having a 6 x 6 Latin square. We ran an extra session when many errors were observed, yielding at least 70 trials per condition.

### Analysis

Participants’ data was excluded when fewer than 20 error-free trials in any single condition were obtained (two in Experiment 1, one in Experiment 2, five in Experiment 3a, and two in Experiment 3b). However, in the remaining participants the average number of error-free trials was much higher (72 in Experiment 1, 54 in Experiment 2, 49 in Experiment 3a, and 69 in Experiment 3b). Most of the errors were due to time-out saccades (5% range [0.2–20], 7% [4–12], 7% [1–15], 2% [0–8], in Experiments 1, 2, 3a and 3b, respectively), followed by choice errors, that is, saccades directed to the wrong location (4% [0.2–10], 3% [1–6], 4% [1–12], 7% [2–13]), anticipations (2% [0–4.9], 3% [2–4], 3% [0–11], 6% [1–16]), and fixation errors (0% [0–1], 3% [1–4], 4% [0–34], 2% [0–12]). The total percentage of errors was 11%, 19%, 18%, and 16% in Experiments 1, 2, 3a, and 3b, respectively. Although the targets had a low contrast to amplify masking effects, they were clearly above detection threshold, as indicated by the generally low percentage of saccades that were made in the wrong direction. In particular, saccades with latencies above 200 ms should give sufficient time to fully process the target and should therefore include only a very tiny number of anticipatory responses. Indeed, when looking at saccades with latencies longer than 200 ms, the percentage of choice errors was even lower, on average 1% (Experiment 1), individually between 0% and 3%, indicating the targets were clearly visible.

We used the R statistics framework (R Core Team, 2014) to test for statistical significance, using *car* and *afex* packages to run repeated measures ANOVAs on median saccadic reaction times. Exponential functions were fitted by minimizing the sum of squared residuals. Optimization was done by using a simplex algorithm (Matlab *fminsearch*).

**Fig. 2:**
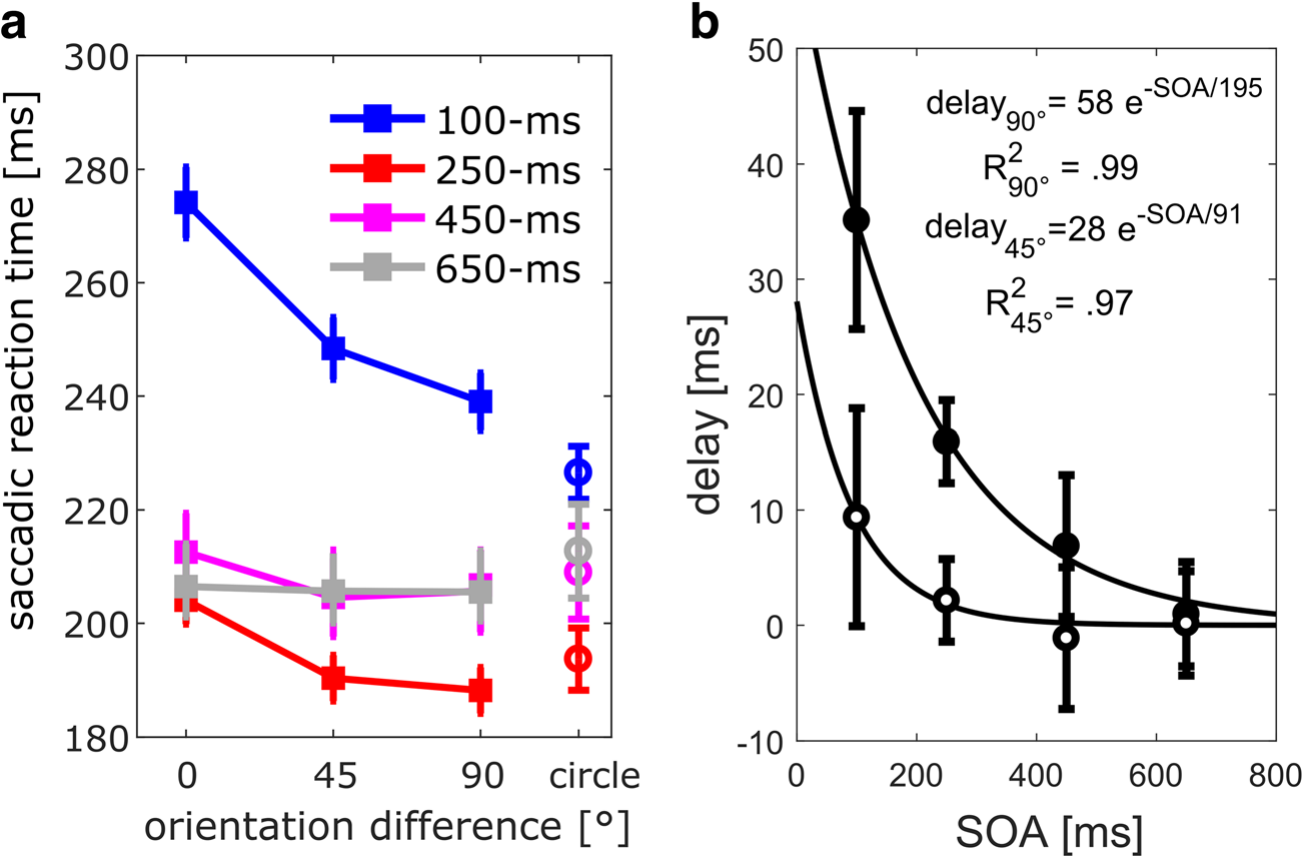
Results from Experiment 1 with bilateral cue presentation and low target contrast. (**a**) Average of median saccadic reaction times (SRTs) as a function of SOA (color-coded) and orientation difference between cue and target. Open circles on the right stand for the circle-only condition (no cue grating). Error bars indicate +/- between-subject standard error of the mean. (**b**) Response delay as a function of SOA. The delay refers to the longer SRTs with 0° relative to 90° cue-target orientation difference (filled circles) and the longer SRTs with 45° relative to 90° cue-target orientation difference (open circles). Error bars indicate 95% confidence intervals (between-subject to facilitate comparison of effects between experiments)

Our main interest was to evaluate the effect of orientation similarity on saccadic reaction times. The effect was found to be well described by exponential decay (Fig. 2b), with delay = *Dmax* · exp(−SOA/*t*), where *Dmax* controls the maximum delay (at time 0) and *t* the exponential time constant, i.e., the time it takes to reach about a third of the maximal delay. Confidence intervals for the exponential function parameters were obtained by bootstrapping (Efron & Tibshirani 1994).

To facilitate comparison of orientation effects graphically within and across experiments we use 95% between-subjects confidence intervals (Cumming, Fidler, & Vaux, 2007). We also report t-tests, assuming unequal variances when comparing accross experiments, with alpha levels corrected for multiple comparisons (Bonferroni).

## Results

### Experiment 1: Bilateral cue presentation

In the first experiment, we assessed whether the repetition of the same orientation would lead to delayed responses to peripheral targets. Importantly, we presented cues on both sides, so that no attention bias should favor responses to one side over the other.

The main findings are shown in Fig. 2a. First, reaction times were about 60 ms longer with the 100-ms SOA compared to other SOAs. Second, saccades were delayed when cue and target had the same orientation (0° orientation difference) compared to the 45° and 90° orientation differences. The effect of orientation repetition decreased exponentially with SOA, as shown in Fig. 2b.

We tested effects of orientation difference and SOA by a repeated-measures ANOVA (3 orientation differences x 4 SOAs) on median saccadic reaction times. We found a main effect of SOA, *F*(3,75) *=* 57.77, *p* < .001, ${\eta}_p^2$ = .70, consistent with an overall reduction in SRT from the shortest to the longer SOAs (259, 198, 213, 209 ms). We found a main effect of orientation difference, *F*(2,50) = 56.70, *p* < .001, ${\eta}_p^2$ = .69, consistent with increasing response times when cue and target had the same orientation, that is, with an orientation difference of zero degrees (0°: 230, 45°: 217, 90°: 212 ms). Importantly, we found an interaction between orientation difference and SOA, *F*(6,150) = 14.56, *p* < .001, ${\eta}_p^2$ = .37, consistent with a rapid decay of the orientation repetition effect with SOA, which we explore below.

#### Forward masking time-course

The delay in reaction times between 0° and 90° orientation difference as a function of SOA was fit by a two-parameter exponential function (cf. data analysis). The bootstrapped parameters indicated a maximal delay of 58 ms at an SOA of 0 ms (*Dmax*; 95% CI 41–81 ms), and a time-constant of 195 ms to reach about a third of the maximal delay (*t*; 95% CI 143–258 ms). Between-subject’s confidence intervals indicate statistically significant delays for SOAs of 100, 250, and 450 ms (see Fig. 2b: error bars do not straddle the 0-delay mark). Fits for the delay in reaction times between 45° and 90° orientation differences indicated a maximal delay of 28 ms (*Dmax*) and a time-constant of 91 ms (*t*). We could not derive reliable confidence intervals for those fits, as a flat line corresponds to infinite *t* values. Accordingly, except for the 100-ms SOA, between-subject’s confidence intervals straddle the 0-delay mark.

The comparison of SRTs with and without grating (i.e., circle+Gabor vs. circle only) gives us an indication of whether there is response suppression that is specific to the presentation of the grating but untuned to orientation. If that was the case we would expect SRTs to be longer for circle+Gabor cues compared to the circle only cues, even for the 90° orientation-difference. Figure 2a shows that overall, SRTs were not significantly longer for circle+Gabor except for the 100 ms SOA (90° vs. circle: 239 ms vs. 226 ms), paired t-test, *t*(25) = 4.67, *p* < .001, Cohen’s d = 1.9.

### Experiment 2: Target contrast manipulation

**Fig. 3:**
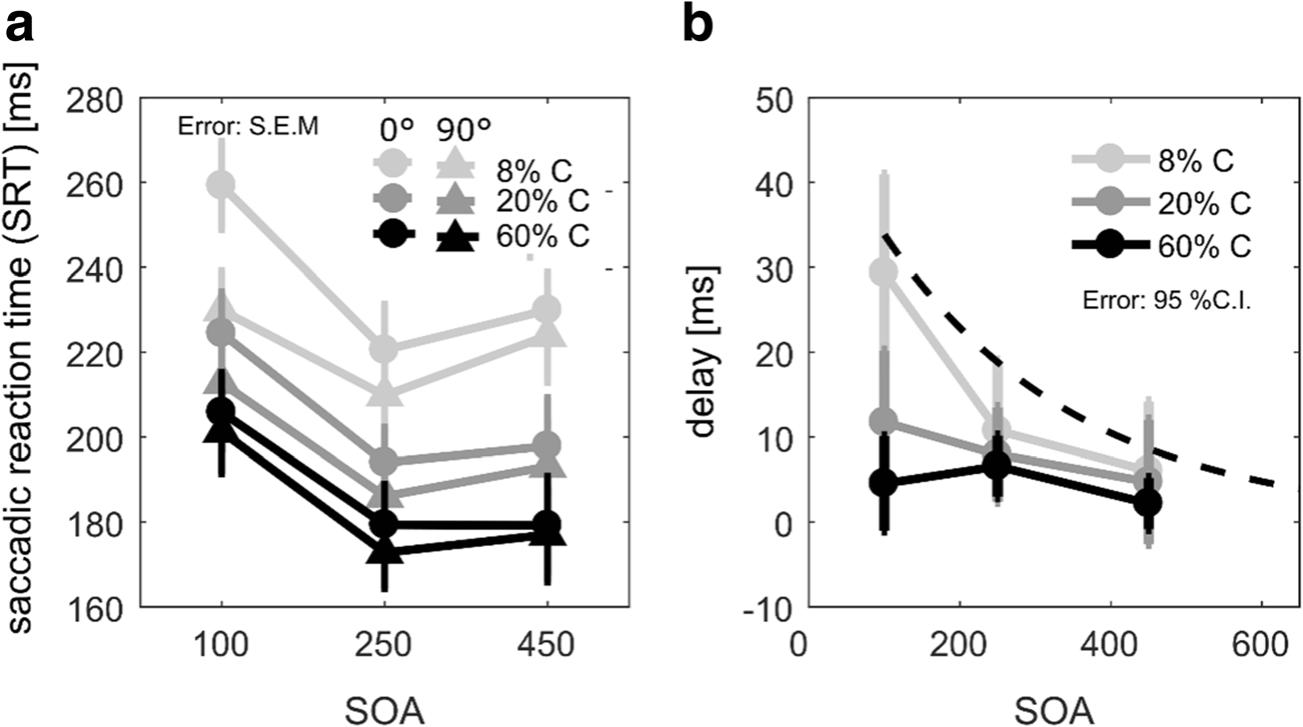
Results from Experiment 2 with bilateral cue presentation and varying target contrast. (**a**) Average of median saccadic reaction times (SRTs) as a function of target contrast, SOA and orientation difference between cue and target. The 0° orientation difference is represented by circles and the 90° difference by triangles. (**b**) The delay of SRTs with 0° compared to 90° orientation difference as a function of SOA and contrasts. The dotted line shows the best fit to the equivalent condition in Experiment 1, for comparison (i.e., 0° vs. 90° orientation difference; see filled circles in Fig. 1b). (**a, b**) Contrast conditions are represented by different shades of gray

Figure 3a-b shows the effect of target contrast and SOA on saccadic reaction times, with cues being presented bilaterally. The lowest contrast of 8% replicates longer SRTs with 0° compared to 90° orientation difference (i.e., the effect of orientation repetition) and the decay of the orientation repetition effect with SOA. The figure also illustrates that target contrast had a prominent effect, reducing SRTs from about 220 ms (8%) to 180 ms (60%). However, target contrast also seemed to greatly reduce the orientation repetition effect. A repeated-measures ANOVA (2 orientation difference x 3 SOA x 3 contrast) confirmed a main effect of SOA (220, 192, 198 ms for SOAs of 100, 250, and 450 ms), *F*(2,24) = 23.17*, p* < .001,${\eta}_p^2$ = .66. It confirmed an effect of contrast, as SRTs were significantly shorter with higher contrasts (227, 200, and 184 ms for 8%, 20%, and 60%), *F*(2,24) = 202.44, *p* < 0.001,${\eta}_p^2$ = .94, reflecting faster information intake (Carpenter, 2004). There was a main effect of orientation difference (209 vs. 199 ms for 0° vs. 90° difference), *F*(1,12) = 77.94, *p* < .001, ${\eta}_p^2$ = .87*,* and an interaction between orientation difference and SOA, *F*(2,24) = 5.40, *p* = .0115, ${\eta}_p^2$ = .31*,* indicating a decrease of the orientation repetition effect with time, just as in Experiment 1. The effect of contrast interacted with orientation, F(2,24) = 10.88, *p* < .001, ${\eta}_p^2$ = .48*,* as suggested by much smaller effects of orientation repetition as contrast was increased (15, 8, and 5 ms for 8%, 20%, and 60%). Finally, the effect of contrast interacted with orientation difference and SOA, *F*(4,48) = 3.21, *p* = 0.021, ${\eta}_p^2$ = .21, which can be appreciated in Fig. 3b, where the response delay of 0° compared to 90° targets is shown for different contrasts and SOAs. The interaction was accounted for by a larger effect of contrast on the effect of orientation repetition at the 100-ms SOA.

### Experiment 3a and Experiment 3b: Unilateral cue presentation

**Fig. 4:**
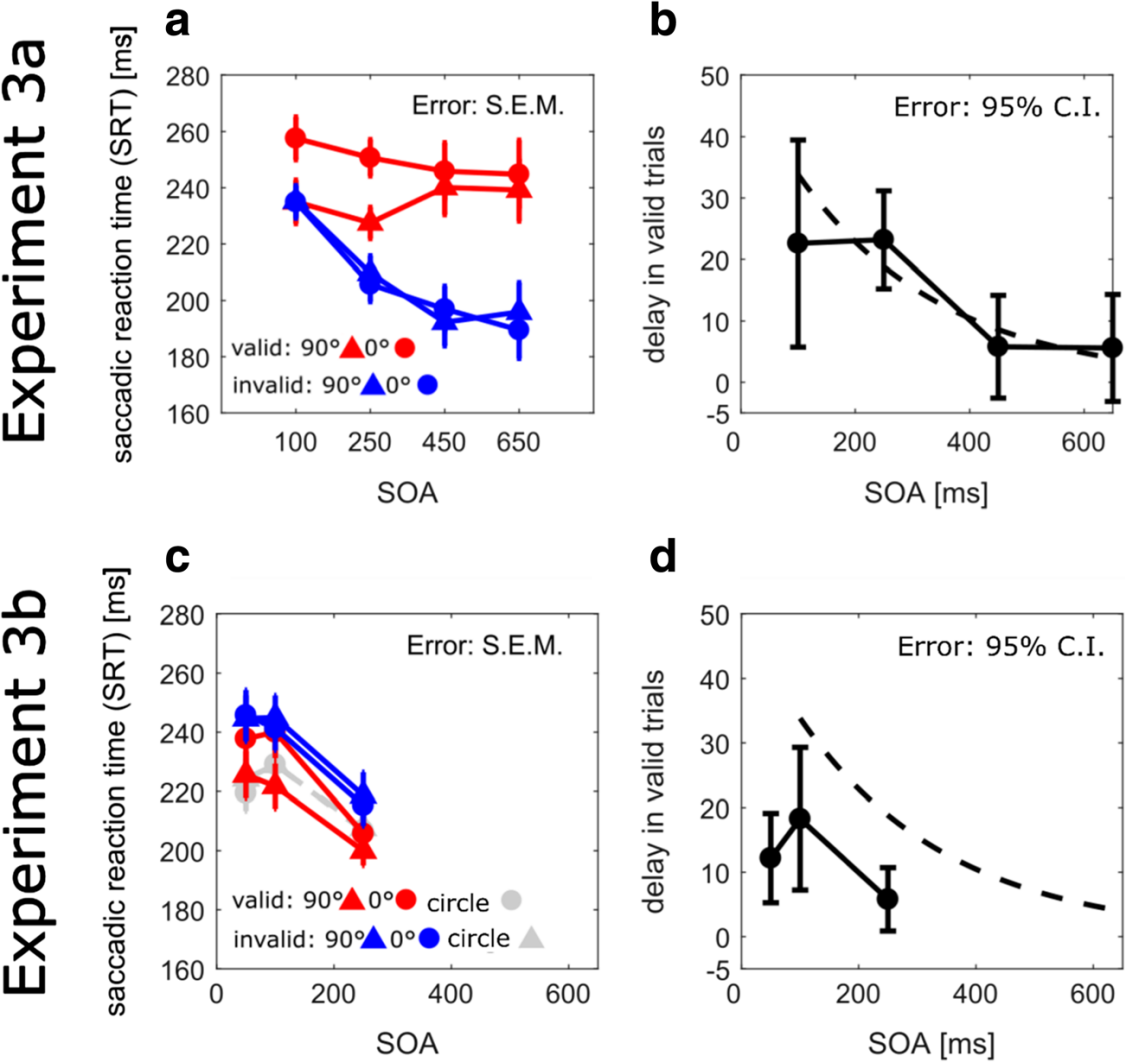
Results from Experiment 3a (**a, b**) and Experiment 3b (**c, d**) with unilateral cue presentation. (**a, c**) Average of median saccadic reaction times (SRTs) as a function of SOA, validity, and orientation difference between cue and target. Circles stand for a 0° orientation difference between cue and target and triangular symbols stand for a 90° orientation difference. Valid trials (i.e., target appearing at the cue location) are shown in red, whereas invalid trials (i.e., target at the uncued location) are shown in blue. (**c**) Data from the circle-only condition are additionally shown in gray, circles stand for valid, triangles for invalid trials. (**b, d**) The delay of SRTs with 0° relative to a 90° orientation difference is shown for valid trials as a function of SOA. The dotted line shows the best fit to the equivalent condition in Experiment 1, for comparison (i.e., 0° vs. 90° orientation difference; see filled circles in Fig. 2b)

Figure 4a-d shows SRTs in Experiments 3a and 3b, in which the cue was presented unilaterally at the same location as the target (valid cue) or at the other location (invalid cue). We wanted to test for an interaction between inhibition of return and forward masking, as indexed by the orientation repetition effect. In Experiment 3b we tested shorter SOAs to elicit facilitation, instead of inhibition of return.

#### Experiment 3a: Interaction with inhibition of return

The comparison of valid (cue and target share the same location) and invalid trials shows an inhibition of return effect: valid cues prolonged SRTs at all but the 100 ms-SOA, where no marked SRT difference between valid and invalid trials was evident (Fig. 4a). Moreover, we still observed an effect of orientation repetition, but only in validly cued trials. The size of the orientation repetition effect is similar to the one obtained with bilateral presentation, as indicated by the overlap of the solid (Experiment 3b) and dashed lines (Experiment 1) in Fig. 4b.

A repeated-measures ANOVA (2 orientation differences x 4 SOAs x 2 validity), confirmed a main effect of SOA, as in previous experiments, with shorter SRTs for longer SOAs (240, 223, 218, and 217 ms for SOAs of 100, 250, 450, and 650 ms), *F*(3,57) = 5.20, *p* = .003, ${\eta}_p^2$ = .21. It confirmed an overall effect of orientation difference, with SRTs being delayed with 0° compared to 90° gratings (228 vs. 221 ms), *F*(1,19) = 10.59, *p* < .01, ${\eta}_p^2$ = .36. The inhibition of return effect was confirmed by an effect of validity, as SRTs were longer at validly cued compared to invalidly cued locations (242 vs. 207 ms), *F*(1,19) = 77.64, *p* < .001, ${\eta}_p^2$ = .80. The effect of validity interacted with SOA, *F*(3,57) = 10.79, *p* < .002, ${\eta}_p^2$ = .36, as expected by inhibition being established within a few hundred milliseconds. More importantly, the effect of validity interacted with the effect of orientation repetition, *F*(1,19) = 21.29, *p* < .001, ${\eta}_p^2$ = .53, showing that the effect of orientation repetition was limited to valid trials where the cue was presented at the same location as the target (249 vs. 235 ms for valid, and 207 vs. 208 ms for invalid). Presentation of cue and target at the same location is a prerequisite for sensory forward masking effects. There was also a significant triple interaction, explained by a decrease of the effect of orientation repetition with time for valid but not invalid trials, *F*(3,57) = 4.82, *p* < .01, ${\eta}_p^2$ = .20. Between-subject’s confidence intervals in Fig. 4b show a significant effect of orientation repetition, for the 100-ms and 250-ms SOA (error bars not straddling the 0-delay mark), following the orientation repetition effects observed in Experiment 1.

#### Experiment 3b: Interaction with facilitation

To see whether attentional facilitation may interact with the orientation repetition effect, we tested shorter SOAs. The main finding shown in Fig. 4c,d was a facilitation effect for all SOAs (i.e., shorter SRTs on valid than invalid trials), even for SOAs that generated inhibition of return in Experiment 3a. We still observed an orientation repetition effect, specifically at the validly cued location (Fig. 4d), but this effect appeared smaller than in Experiment 3a (Fig. 4b).

A repeated measures ANOVA (2 orientation difference x 3 SOA x 2 validity) showed a main effect of SOA, *F*(2,42) = 25.79, *p* < .001, ${\eta}_p^2$ = .55, and a main effect of orientation difference, *F*(1,21) = 18.42, *p* < .001, ${\eta}_p^2$ = .47. As in previous experiments, SRTs were overall shorter for longer SOAs (238, 237, and 209 ms for SOAS of 50, 100, and 250 ms). Orientation differences of 0° led to overall longer SRTs than 90° differences (231 vs. 226 ms). There was also an interaction between orientation difference and validity, *F*(1,21) = 18.55, *p* < .001, ${\eta}_p^2$ = .47, as the orientation repetition effect was only present at the validly cued location (valid: 12 ms, invalid: -1 ms). No further effect or interaction reached significance.

As in Experiment 1, we included a condition in which only the circle was shown. However, unlike in Experiment 1, we controlled for a possible oddball effect by presenting the circle-only condition in separate blocks. Circle-only trials gave nearly identical SRTs in valid and invalid conditions (with a maximal difference of 3 ms for the 100-ms SOA. Moreover, in invalid trials, circle+Gabor cues gave longer SRTs (235 ms) than circle-only cues (220 ms), paired t-test, *t*(21) = 4.85, *p* < .001, d = 2.04. This pattern of results may indicate that the circle alone was not an effective cue in capturing attention, especially in the blocked design, possibly because observers were set to look for an object that did not resemble the circle (i.e., a Gabor). It is known that peripheral cueing effects depend on cue-target similarity (Folk, Remington, & Johnston, 1992).

#### Comparison between Experiments 3a and 3b

A comparison of data in Figs. 4b and d suggests that the orientation repetition effect was not the same across experiments. We compared SRTs with SOAs of 100 and 250 ms, which were both tested in Experiments 3a and Experiment 3b, but resulted in opposite effects of cue validity (IOR vs. facilitation). The orientation repetition effect was smaller when attentional facilitation was observed in Experiment 3b (SOA of 100 ms: 18 ms [CI 7–29]; SOA of 250 ms: 6 ms [1–11]) compared to Experiment 3a (SOA of 100 ms: 23 ms [CI 6–39]; SOA of 250 ms: 23 ms [15–31]). A statistically significant difference between Experiments 3a and 3b was confirmed for the 250-ms SOA, *t*(32.152) = 3.881, *p* < .001, d = 1.34. In Experiment 3b, where we would expect the largest orientation effect with the shortest SOA of 50 ms, we observed a much smaller one (12 ms [5–19]) than predicted by the exponential fit to orientation-difference effects in Experiment 1 (45 ms [29–64], with bootstrapped confidence intervals).

#### Error analysis

We looked for a possible speed-accuracy tradeoff by analyzing directional errors. As mentioned above, the proportion of directional errors was overall small (< 7%). Its distribution had a strong positive skew. The modal value of the error rates was 0 in all experiments, meaning there were no choice errors under most conditions. Overall, error rates indicated no trade-off as they followed the pattern of SRTs. For brevity, we only describe the pattern of mean error rates and forgo a full report of the statistical analyses. In Experiment 1, error rates were largest (6%) with the shortest SOA and dropped to less than 1% with the longest SOA. Further, error rates were similar across orientation difference conditions (3% for 0°, 2% for 45°, and 4% for 90°). In Experiment 2, we observed the same effects. Additionally, error rates decreased slightly with increasing target contrast (from 3% at the lowest contrast to 2% at the highest contrast). The effect of orientation repetition was at most an increase of 1% in error rates (3% for 0° and 2% for 90° with a 8% target contrast). In Experiment 3a, error rates depended mainly on SOA (11% with the shortest SOA compared to 1% with the longest) and orientation difference in valid trials (valid: 2% for 0° vs. 1% for 90°). Error-rate was 3% in invalid trials. In Experiment 3b, we observed similar error rates for the range of SOAs (4–6%), but a strong effect of validity, with error rates being much lower for valid cues (< 1%) compared to invalid cues (< 12%). There was little effect of orientation repetition on either valid or invalid trials (differences < 0.5%). Therefore, the error analysis indicates no speed-accuracy tradeoff, which could have indicated the influence of decisional factors on SRTs. In contrast, a delay in sensory processing is expected to have similar effects on the ability to respond promptly to the target presentation and the ability to make correct decisions, which is what we observed.

## Discussion

We tested the effect of forward masking on saccadic reaction times by presenting cue and target gratings of varying orientation in close succession. In the first two experiments, we presented cues on both sides of fixation, measuring forward masking in the absence of a spatial orienting bias. In a third experiment, we presented cues unilaterally, measuring the interaction between forward masking and cueing effects known as attentional facilitation and IOR (Posner & Cohen, 1984).

With bilateral cue presentation, we observed a substantial orientation repetition effect of about 30 ms with short SOAs (100 ms). Saccades were delayed when target and cue had the same orientation compared to differently oriented cue-target pairs. This effect waned exponentially across time, disappearing within 500 ms after cue onset. Consistent with forward masking, the orientation repetition effect was greatly reduced when the target contrast was high (Experiment 2): Effects were large at low target contrast (8% Michelson contrast), but greatly reduced at a contrast of 20%, and nearly absent with a 60% contrast.

Importantly, with low target contrasts and with cues presented unilaterally, the orientation repetition effect only occurred when cue and target were presented at the same location (valid trials) and contributed to the IOR effect (SRTs being slower at validly cued locations) in an additive way (Experiment 3a-b). For SOAs that generated an IOR effect, the orientation repetition effect was of the same size as with bilateral cues. On the other hand, an experiment that generated a facilitation effect (SRTs being faster at validly cued locations; Experiment 3b) produced a smaller orientation repetition effect, indicating attenuation of forward masking by attentional orienting.

Contrary to the initial view that IOR is the consequence of reorienting attention after being captured by the cue, facilitation and IOR can be better characterized as originating from independent processes with different time-courses (Berlucchi, Chelazzi, & Tassinari, 2000; Danziger & Kingstone, 1999; Klein, 2000; Lupiáñez, 2010; Tassinari, Aglioti, Chelazzi, Peru, & Berlucchi, 1994). Consistent with the latter, facilitation was found to affect temporal order judgements, whereas IOR did not (Gibson & Egeth, 1994). Our experiments lend further support to the view that IOR does not affect the sensory stage of responding to a peripheral target, but a decisional stage (Ludwig, Farrell, Ellis, & Gilchrist, 2009), by showing that facilitation but not IOR attenuates forward masking. Facilitation of saccadic reaction times may be observed because attention is drawn towards the cued location, allowing to individuate cue and target events, equivalent to increasing the target contrast. This is supported by the effect of attention on contrast sensitivity and physiological recordings showing that the benefit of attention on the contrast response of visual cells is equivalent to an increase in effective contrast (e.g., Buracas & Boynton, 2007).

We attribute the orientation repetition effect to forward pattern masking. We propose that forward masking is caused by a sensory adaptation mechanism and not by object updating costs. Consistent with a sensory origin, the effect waned rapidly and was greatly reduced when target contrast was increased. Carpenter (2004) showed that sensory factors are negligible in determining saccadic reaction time for high contrast targets, compared to decisional factors determining the time it takes the oculomotor system to commit to one option over the other. For low contrast targets, however, the slowing down in the accumulation of sensory evidence by forward masking can have a substantial effect on saccadic reaction times.

Incidentally, we observed facilitation in one experiment but inhibition of return in another for the same SOAs. The only difference lies in the range of SOAs experienced by the participants in the different short blocks of trials. Inhibition of return has been shown to be sensitive to temporal statistics, with a larger IOR in more volatile environments (Farrell, Ludwig, Ellis, & Gilchrist, 2010). Therefore, the transition between facilitation and inhibition of return may vary depending on the range of SOAs presented.

Previous studies failed to find an effect of orientation repetition in IOR paradigms. Kwak and Egeth (1992) tested not only color, but also the effect of same and different orientations on IOR with SOAs of about 700–1,300 ms and a cue of 300-ms duration. The paradigm succeeded in producing a robust IOR effect. But like their color results, they found no evidence of orientation repetition. Rather, a small facilitation effect for same-orientation irrespective of location was observed. Another study found no effect of orientation similarity with a limited range of SOAs (100 and 500 ms; Higenell, White, Hwang, and Munoz, 2013). In both studies, the SOAs chosen for measuring IOR were in a range where our orientation repetition effects were already greatly reduced. Further, in Higenell et al. (2013) it is likely that the effect of forward masking was absent at the short SOA because facilitation counteracted forward masking. Indeed, we have shown that forward masking is reduced when spatial facilitation is observed.

### Forward masking mechanism

The orientation repetition effect we observed, where saccades are delayed when cue and target have the same orientation and are presented at the same location, can be explained by rapid adaptation in orientation-selective cortical cells (for a review, Kohn, 2007). Perceptually, adaptation results in a reduction of visibility that can be compensated for by increasing the test stimulus strength. Psychophysical adaptation experiments typically show the adapter stimulus for tens of seconds, but adaptation aftereffects have occasionally been reported for adapter durations of milliseconds (Sekuler & Littlejohn, 1974; Suzuki, 2001). Likewise, physiological studies report rapid adaptation in orientation-tuned cortical neurons, in the form of reduced response rates (Muller, Metha, Krauskopf, & Lennie, 2001; Nelson, 1991). More recently, a motion aftereffect and adaptation of the response rate of MT neurons was measured after adapter durations of 25 ms (Glasser, Tsui, Pack, & Tadin, 2011).

Rapid adaptation has been related to contrast gain control, a functional mechanism by which neurons adjust their dynamic range to the prevailing image contrast, avoiding response saturation and thus maximizing the transmission of information (Carandini & Heeger, 2012; Crowder, Hietanen, Price, Clifford, & Ibbotson, 2008; Geisler & Albrecht, 1992; Hu, Wang, & Wang, 2011; Ohzawa, Sclar, & Freeman, 1982). The effect of the adapter is to reduce the effective contrast of the stimulus, equivalently to a shift in the contrast response function of the neuron (Crowder et al., 2008; Hu et al., 2011; Ohzawa et al., 1982). Unlike other forms of adaptation, contrast gain control is fast, and ensures a rapid adjustment to local contrast over space and time. It could be caused by a process of divisive normalization of a neurons’ response by inhibition from other neurons within its network (Carandini & Heeger, 2012). This mechanism may account for contrast adaptation with short adapter durations (Greenlee, Georgeson, Magnussen, & Harris, 1991). Here, the cue would shift the contrast response of neurons tuned to the cue orientation for a short time, causing a delay in processing the target stimuli sharing the cue orientation, compared to differently oriented targets.

### Consistence with previous studies

Although contrast gain control can explain our orientation repetition effects on saccadic reaction times, previous studies, showing feature repetition effects with high contrast shapes, may require higher-level explanations (Patel et al., 2010). In those cases, explaining a sizable portion of the repetition effect by contrast gain control alone may require a reduction in the target’s effective contrast that would be unrealistic (Carpenter, 2004). One of such higher-order mechanisms could be object updating costs (Fox & de Fockert, 2001; Lupiáñez, 2010). The object file theory has been postulated as a mechanism to maintain object continuity in space and time (Kahneman, Treisman, & Gibbs, 1992). For instance, two blinking lights can be seen as the same object moving back and forth. In that case the light is integrated into a single object file. This could explain priming effects, where an object file is opened by a prime and facilitates recognition of the object on the second instance. In the context of IOR, integration into the same object file can be detrimental to detecting the object as a novel event rather than a continuation of the previously presented one, entailing an “onset detection cost” (Lupiáñez, 2010). Those object updating costs could be implemented by mutual inhibition between shape representations in the parietal cortex (Patel et al., 2010; Red et al., 2012). Object updating is less likely to explain our results, as we did not change shape similarity but orientation. Here, we favor an interpretation of the cue-target similarity effects on saccadic reaction times at the feature-level. We explain those effects as a consequence of the adaption of orientation sensitive neurons to repeated stimulation by a mechanism of contrast gain control.

## Conclusion

We show that forward masking, likely due to rapid contrast gain control, can delay saccade reaction times after a time-period that is much longer than initially assumed. Forward masking contributes to the IOR effect in an additive manner, whereas it is reduced by attentional orienting towards the target location, showing that facilitation and the following inhibition of motor responses are due to separate factors.
